# Equivalent method for obtaining concrete age on the basis of electrical resistivity

**DOI:** 10.1038/s41598-021-00447-8

**Published:** 2021-11-05

**Authors:** Xiaochun Lu, Fuguo Tong, Xinyuan Zha, Gang Liu

**Affiliations:** College of Hydraulic & Environmental Engineering, China Three Gorges University, Yichang, 443000 China

**Keywords:** Civil engineering, Structural materials

## Abstract

Concrete age is the time since the moment water is added to the cement, and the age of concrete comprehensively reflects the physical properties of the concrete when curing under standard conditions. For concrete under nonstandard conditions, its physical properties are directly related to both its age and temperature history. The equivalent age of concrete is the time at which concrete under nonstandard conditions reaches the same state as concrete under standard conditions. Most equivalent methods, such as the Nurse-Saul function and the Arrhenius function, are based on a maturity index. However, the accuracy of these methods breaks down when the curing temperature range is wide. In this paper, the electrical resistivity of concrete is used as the index to determine the equivalent age of concrete. This method is based on the assumption that concrete with the same mixture proportions has the same electrical resistivity when the maturity of the concrete is the same, regardless of the curing history. The proposed method is advantageous because it can be performed in real time and is nondestructive. To constantly measure the electrical resistivity of concrete, an automatic data acquisition system is developed to monitor the electrical resistivity of concrete and reduce the error caused by polarization as much as possible. Then, a model for predicting the electrical resistivity of concrete under different curing temperatures is proposed to conveniently calculate the equivalent coefficient. Finally, the results calculated by the proposed equivalent method are compared with those of the standard method (Nurse-Saul equation), and the results of the proposed model are found to be more reasonable.

## Introduction

Concrete is widely used in civil buildings and water conservation projects because of its good mechanical performance^1,2^. The mechanical performance of concrete is an important factor that affects the progress of engineering, the safety of buildings, and the state of service^2–4^. Generally, concrete age is used to comprehensively reflect the physical and mechanical properties of concrete curing under standard conditions. For concrete curing under nonstandard conditions, the equivalent age is used to evaluate the state of the concrete. Concretes with the same composition will have the same strength when they have the same equivalent age, even with different curing histories. Identifying concrete equivalent age is a convenient way to determine the state of concrete^5,6^. For standard curing conditions (20 °C, RH ≥ 95%), the concrete equivalent age equals its actual age. However, under natural conditions, the concrete curing environment differs considerably from the standard curing environment^7^. Therefore, obtaining the equivalent age through an age equivalent method is often necessary.

Concrete age equivalent methods can typically be classified into three types: direct, semi direct and indirect methods. The direct methods mainly involve taking cores of the structure to test the compressive strength to obtain the equivalent age. However, for many critical structural parts, drilling is not allowed. The semi direct methods mainly involve measuring the strength of samples cured under the same conditions as the structure; thus, the structure has the same age as the reserved samples^8,9^. The obvious disadvantage of this method is that the number of test blocks that need to be reserved should be as large as possible because the compressive test experiment is a nonrepeatable fracturing experiment, and the test data points are relatively discrete; furthermore, the performance of a test block cannot represent the performance of the building structure^6,10,11^. The indirect equivalent methods establish a correlation between concrete age and the physical quantities of concrete, such as the elastic modulus and rebounding value. After measuring the relevant physical quantity, the equivalent age of the concrete can be calculated in accordance with the standard relation curve, which is obtained under standardized curing conditions. Indirect testing methods include the pull-out test^12–15^, rebound^16^, ultrasonic^17^, ultrasound–rebound synthesis^18,19^, and maturity methods^20–24^. The indexes used in the indirect test methods differ, but the equivalent concrete age can be obtained through conversion. The principle is that macroscopic physical properties are determined by the internal materials and pore structure of the concrete. However, existing indirect testing methods still demonstrate shortcomings in terms of the test time, scope, and accuracy. For example, the pull-out test method can be used when the concrete has a minimum strength, and the rebound method can only accurately measure changes in the surface most 10–15 mm of the concrete^25,26^. On the basis of the principle of indirect methods, this paper presents a method for obtaining the equivalent age of concrete via electrical resistivity.

Concrete is generally believed to be a poor conductor or insulator of electricity, but in reality, this property is not absolute^27,28^. The electrical conductivity of concrete is related not only to its composition but also to its pore solution content, temperature and age^29–31^. Electrical resistivity is the opposite of electrical conductivity^32^. The resistivity variation range of ordinary concrete is generally between 10 and 10^12^ Ω m^33,34^. Whittington et al.^35,36^ and Sun et al.^37^ reported that concrete conduction can be divided into ionic, electronic, and hole conduction. Concrete electrical resistivity has been widely used in many areas. In the early 1930s, Petin and Gajsinivitch^38^ studied the hydration process of cement by measuring the conductivity of concrete to establish the correlation between concrete resistivity and cement hydration. Concrete resistivity has also been used in chloride ion permeability detection^39^, damage detection^40,41^, and oxygen diffusion^42^. The resistivity of concrete is determined by the material characteristics and pore structure of concrete^43,44^. In theory, the electrical resistivity of concrete should be the same for concrete with the same composition and structure but different curing histories.

This study proposes an equivalent method for determining concrete age by using concrete resistivity. First, the standard evolution curve of concrete electrical resistivity is obtained under standard curing conditions. Then, the electrical resistivity of concrete is tested under nonstandard curing conditions, and a model for predicting the electrical resistivity of concrete is presented. Finally, the equivalent method based on electrical resistivity is presented and compared with the maturity method. Through the comparison, the proposed equivalent method can quickly determine the actual concrete age in real time and is more reasonable than the standard method.

## Experimental test on concrete electrical resistivity

The testing methods for concrete resistivity are mainly divided into two-, four-, and no-electrode methods^45–48^. In accordance with the purpose and requirements of this test, the direct current (DC) diode method with two electrodes is selected to measure the electrical resistivity of concrete.

### Testing system for concrete resistivity

To obtain the electrical resistivity of concrete, an automatic concrete resistivity acquisition system is designed. The system can test the electrical resistivity of concrete quickly, accurately and in real time and consists of a data acquisition device, automatic control system and temperature control system.

The data acquisition device is an intelligent resistivity meter that can automatically adjust the test range of resistance to ensure the accuracy of the result. The meter communicates with a computer, and the frequency of data collection can be programmed. The measured data can be stored in the computer, and specific indicators of the device are shown in Table 1.

**Table 1:** Specific indicators of the intelligent resistivity meter.

Measuring range	20 mΩ	200 mΩ	2 Ω	20 Ω	200 Ω	2 kΩ	20 kΩ
Accuracy	0.2% ± 3			0.1% ± 3			
Testing current	100 mA	100 mA	100 mA	10 mA	1 mA	100 uA	100 uA
Resolution ratio	10 uΩ	10 uΩ	100 uΩ	1 mΩ	10 mΩ	100 mΩ	1 Ω
Open-circuit voltage		< 1.0 V			< 5.0 V		

**Figure 1:**
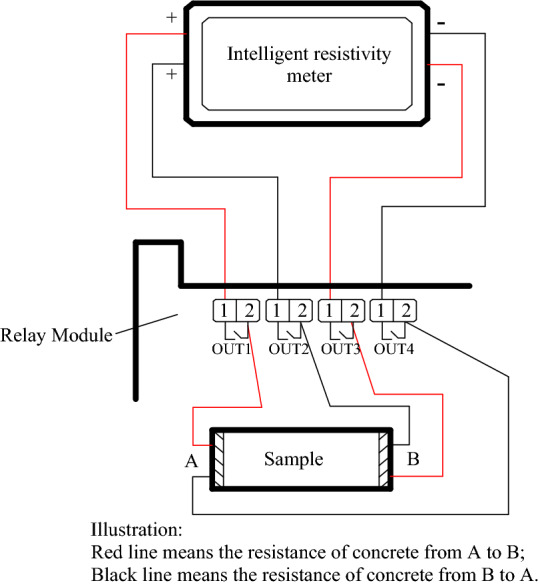
Working principle of the automatic concrete resistivity acquisition system.

The automatic control system is used to reduce the effect of polarization caused by the DC test. According to previous research, during concrete testing, concrete exhibits the polarization phenomenon, which leads to a negative resistance value in one direction^49,50^. Despite this condition, the resistance values of the concrete in both directions are relatively stable. Therefore, the average of the resistance values in two directions in the concrete is used to represent the overall resistivity. This usage requires the test system to automatically implement reverse measurements. In this test, multiple relay modules are used to control the opening and closing of acquisition lines for the automatic switching of the resistance test direction. The working principle is shown in Fig. 1.

Forward resistance of sample collection arises when relay 1, port 3 is closed; reverse resistance of sample collection emerges when relay 2, port 4 is closed. In the PC terminal, relay port opening and closing can be achieved through program control via automatic reverse acquisition by the resistance meter. Although the resistance in both directions was tested, the polarization of the concrete could not be eliminated. Therefore, a resistor box with a large resistance is connected in series to the circuit, thereby preventing the measurement of a negative resistance value by the intelligent resistivity meter. By adopting certain technical measures, the problem of concrete polarization caused by the two-electrode method can be eliminated as much as possible. The temperature control system is mainly used to control the concrete temperature during curing and to keep the curing temperature constant.

### Concrete electrical resistivity tests at different temperatures

#### Test samples

**Figure 2:**
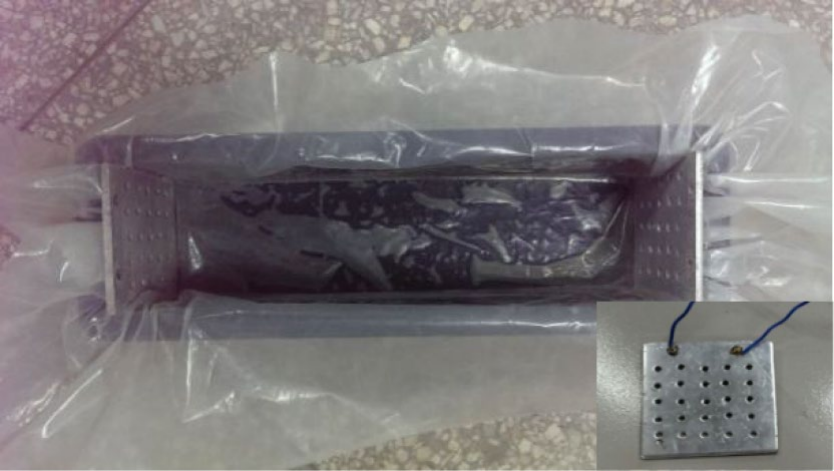
Test using a plastic film and the electrode.

**Figure 3:**
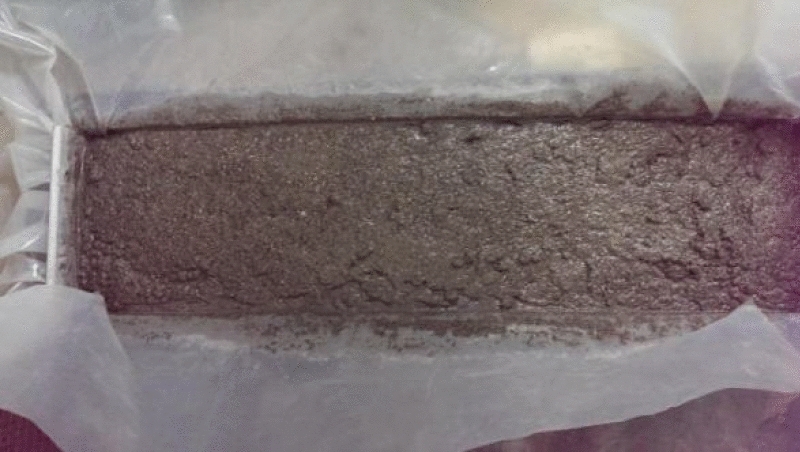
Surface of the sample after vibration.

The sample size in the test is 100 × 100 × 400 mm. The measuring electrode is composed of an aluminum plate with a size of 100 × 100 × 3 mm, in which boreholes are evenly drilled to increase the contact area between the concrete and the electrode (Fig. 2). Measuring electrodes are placed on both ends of the sample, and the concrete sample and measuring electrodes are placed in the mold until the end of the test. In this test, composite Portland cement (Huaxin brand), which is rated 32.5 MPa, is used. The water-cement-sand ratio is 1:2:6.25. According to the national standard “Standard for test method of mechanical properties of ordinary concrete (GB/T 50081-2002)”, the cement, water, and sand are fully mixed and transferred into the mold. Then, the mixture is vibrated on a shaking table for 10 min until the concrete surface shows bleeding (Fig. 3).

#### Testing scheme

The evaluation of concrete resistance with time under different curing temperatures is measured in this test. When designing the test scheme, the following factors should be the same: the water-cement ratio, the proportion of sand, the sample size, the curing humidity, and the vibratory compactness of the concrete. Only the curing temperature and the curing time are changed. Considering that the temperature of the concrete service environment is generally between 20 and 50 °C, the curing temperatures are set to 20 °C, 25 °C, 30 °C, 35 °C, 40 °C, 45 °C, and 50 °C. GDS-408 constant temperature and humidity control box is used to control the test temperature. Because the volume of the sample is relatively small, the increase in temperature because of hydration can be ignored.

### Testing results

**Figure 4:**
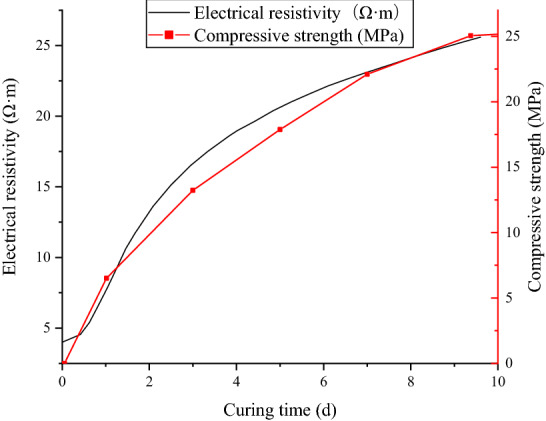
Resistivity growth curve and compressive strength curve of concrete under curing conditions of 20 °C.

The electrical resistivity of concrete changes continuously as concrete hydrates. The evaluation of the concrete electrical resistivity under standard curing conditions (20 °C, 95% RH or greater) is tested first. Additionally, we also test the compressive strength of concrete under standard curing conditions. The resistivity growth curve and the compressive strength curve are shown in Fig. 4. The compressive strength is obtained by the standard cube test^51^.

Figure 4 shows that with the increase in curing time, the concrete resistivity increases gradually and shows the same tendency as the increase in the compressive strength. After the hydration reaction begins, the resistivity decreases sharply within a short time, followed by a rapid increase. This rapid increase is primarily concentrated in the range of 0.1–0.3 days. After a period of rapid growth, the growth rate of resistivity gradually slows down and stabilizes. The variation pattern of concrete resistivity reflects the reaction process of the concrete itself. In the initial stage of the reaction, the cement is dissolved in water, and the amount of conductive ions in the concrete increases rapidly, leading to an enhanced reaction. As the reaction proceeds, water is continuously consumed by cement condensation and hardening, resulting in a continuous decrease in the total amount of ions involved in electricity conduction, thus increasing the resistivity of the concrete. After the cement is fully dissolved, the contact area between the cement particles and the water widens, the hydration reaction is sufficient, and the water consumption increases, leading to a sharp increase in the resistivity stage. With the continuous generation of hydration products, the surface of the cement particles becomes coated, leading to a decrease in the reaction rate. Thus, the resistivity growth rate decreases. Wei et al.^33^ studied the hydration of cement by using the resistivity method, and they divided hydration into three stages: dissolution, induced formation, and setting and hardening. The variation pattern of concrete resistivity in this study is consistent with that reported by Wei et al.^33^.

**Figure 5:**
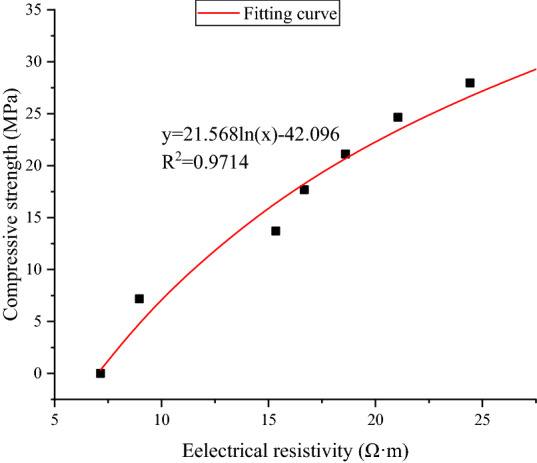
Relationship between the electrical resistivity and the compressive strength.

It can be found that the compressive strength of concrete has a correlation with the electrical resistivity of the concrete under standard curing conditions^52^. Generally, the higher the electrical resistivity of concrete is, the higher the compressive strength is. The reason for this correlation is that both the compressive strength and the electrical resistivity of concrete reflect the state of the concrete. The relationship between electrical resistivity and compressive strength is shown in Fig. 5, where the horizontal axis is resistivity, and the vertical axis is compressive strength. A logarithmic function is used for fitting the relationship between the electrical resistivity and the compressive stress. From this curve, if we know the electrical resistivity of the concrete, we can conveniently obtain the compressive strength of the concrete.

**Figure 6:**
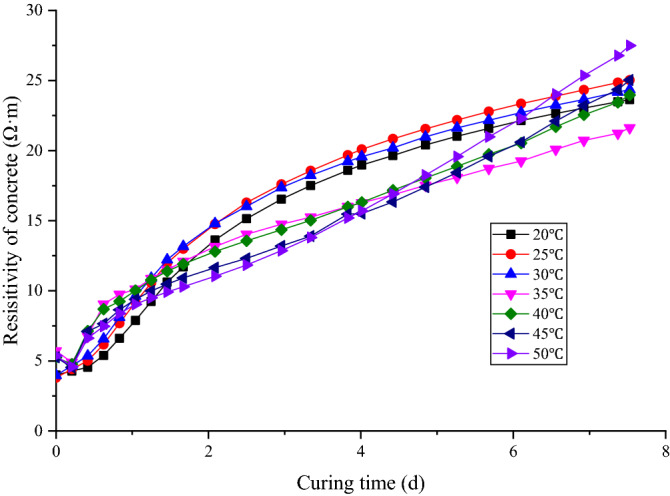
Evolution of concrete electrical resistivity under different curing temperatures.

According to the test scheme, the electrical resistivity of concrete under different curing temperatures was tested. Considering the large amount of data collected in the experiment, data at fixed time intervals are extracted for processing without affecting the regularity of concrete resistivity change. The main experimental results are discussed below (Fig. 6).

The concrete resistivity data at different curing temperatures are compared in Fig. 6. The results show that concrete resistivity does not have a simple positive linear relationship with curing time but presents a complex nonlinear change. The curing temperature has a considerable influence on the concrete resistivity. In the initial stage of concrete mixing, the concrete resistivity at different curing temperatures exhibits a small difference and a trend with an initial decrease followed by an increase. As the reaction progresses, the concrete resistivity increases with increasing curing time. In general, the higher the temperature is, the faster the concrete resistivity increases. At higher temperatures, the hydration process is faster. Thus, the electrical conductivity decreases more rapidly. The electrical conductivity of a material (in a fixed phase) is also lower at higher temperatures. However, when the curing time reaches approximately 2 days, the resistance trends of the concrete samples differ. When the curing temperature is increased from 20 to 30 °C, the higher the temperature is, the greater the resistivity value is. When the curing temperature is increased from 30 to 50 °C, the higher the temperature is, the lower the resistivity value is. When the curing time is approximately 6 days, the variation patterns of concrete resistivity show obvious differences; that is, the concrete resistivity under high-temperature curing is higher than that under low-temperature curing. In this test, the main factors that affect concrete resistivity are the temperature and the curing time. Theoretically, the higher the temperature is, the lower the concrete resistivity is; the longer the curing time is, the higher the concrete resistivity is. However, the experimental data show that the effects of temperature and curing time are most prominent at different times.

As an anonymous reviewer mentioned, this phenomenon can be explained from the perspective of ionic mobility inside the concrete pores. The reason why early age concrete can conduct electricity is that there are many mobile ions in its voids. The increase of temperature will accelerate the movement of ions in the solution, thus showing stronger conductivity. But on the other hand, the temperature increase will accelerate the hydration reaction of concrete, making the connectivity between the pores of concrete become worse, and the ion cannot complete the movement from one electrode to the other electrode, thus increasing the resistivity of concrete. Therefore, the change caused by the increase of temperature is two aspects. On the one hand, it is conducive to enhance the conductivity of concrete, and on the other hand, it is not conducive to the increase of concrete conductivity. Therefore, the resistivity of concrete shows different laws in different temperature ranges.

## Equivalent method of concrete age

### Model for predicting the resistivity of concrete

The use of concrete resistivity for determining the equivalent concrete age is derived from the traditional method that uses a maturity index to obtain the equivalent age of concrete. Whether concrete resistivity or another index is used, the equivalent age of concrete is determined by the concrete’s internal materials, pore structure, and other microstructures. Therefore, in the proposed equivalent method, the resistivity of concrete can be used as an equivalent index.

Concrete can be composed of a solid skeleton and a gas–liquid mixture filling the pores of the solid skeleton. The resistivity of concrete is affected by that of the solid skeleton, that of the gas–liquid mixture, and the proportion of the two substances. According to the Wiener bounds theory^53,54^, the upper and lower bounds of concrete resistivity are obtained when the solid and gas–liquid mixture are in series or parallel. Therefore, the resistivity of concrete can be expressed as follows:

1 $$\rho =\left(1-\eta \right)\left({\rho }_{s}+{\rho }_{m}\right)+\eta \left(\frac{{\rho }_{s}{\rho }_{m}}{{\rho }_{s}+{\rho }_{m}}\right),$$

where $\rho$ is the concrete electrical resistivity; ${\rho }_{s}$ is the resistivity of the solid phase; ${\rho }_{m}$ is the resistivity of the water–gas mixture; and $\eta$ is the proportion of the series arrangement, which differs among concretes with the different porosities and can be expressed as a function of porosity. Considering that the porosity of the concrete changes with the hydration of the concrete, the parameter $\eta$ can be expressed as a function of concrete age (starting when water is added to the cement) and curing temperature. As concrete gradually hardens, the proportion of water content in the solid skeleton pores changes. The resistivity of a mixture of water and air can be expressed as follows:

2 $${\rho }_{m}=\left(1-\alpha \right)({\rho }_{g}+{\rho }_{l})+\alpha \frac{{\rho }_{l}{\rho }_{g}}{{\rho }_{g}+{\rho }_{l}},$$

where $\alpha$ is the proportion of the series arrangement, which can be expressed as a function of the water volume fraction; ${\rho }_{l}$ is the pure water resistivity; and ${\rho }_{g}$ is the resistivity for pure air. When the temperature is 20 °C, ${\rho }_{l}=238$ ${\Omega{\text{m}}}$ and ${\rho }_{g}=1\times {10}^{13 }{\Omega{\text{m}}}$. Similarly, the volume fraction of water in the pores varies with the hydration of concrete; therefore, parameter $\alpha$ can be expressed as a function of concrete age and curing temperature.

According to comprehensive Eqs. (1) and (2), the resistivity of concrete can be expressed as follows:

3 $$\rho =\left(1-\eta \right)\left[{\rho }_{s}+\left(1-\alpha \right)\left({\rho }_{g}+{\rho }_{l}\right)+\alpha \frac{{\rho }_{l}{\rho }_{g}}{{\rho }_{g}+{\rho }_{l}}\right]+\eta \left[\frac{\left(1-\alpha \right)\left({\rho }_{g}+{\rho }_{l}\right){\rho }_{s}+\alpha \frac{{\rho }_{l}{\rho }_{g}{\rho }_{s}}{{\rho }_{g}+{\rho }_{l}}}{{\rho }_{s}+\left(1-\alpha \right)\left({\rho }_{g}+{\rho }_{l}\right)+\alpha \frac{{\rho }_{l}{\rho }_{g}}{{\rho }_{g}+{\rho }_{l}}}\right].$$

Analysis of Fig. 5 indicates that the influence of temperature on concrete resistivity is complex. The items in Eq. (3) are analyzed to obtain a specific expression for concrete resistivity.

1. The electrical resistivity of air is larger than that of water or solids, and the ratio of water or solids to air can be neglected, such as $\frac{{\rho }_{l}}{{\rho }_{g}}\approx 0$ and $\frac{{\rho }_{s}}{{\rho }_{g}}\approx 0$. Therefore, Eq. (3) can be expressed as:
  4 $$\rho =\left(1-\eta \right)\left[{\rho }_{s}+\left(1-\alpha \right)\left({\rho }_{g}+{\rho }_{l}\right)+\alpha {\rho }_{l}\right]+ \eta \left[\left(1-\alpha \right){\rho }_{s}\right] =\left(1-\alpha \eta \right){\rho }_{s}+\left(1-\eta \right)\left(1-\alpha \right){\rho }_{g}+\left(1-\eta \right){\rho }_{l}$$
2. According to the test results, the resistivity of concrete shows a logarithmic relationship with time, and the relationship with temperature can be expressed by cubic function. In addition, the curing time should be corrected by temperature. Therefore, the resistivity of solid phase can be expressed as a function of temperature and a function of age after temperature correction. Considering that the electrical resistivity of concrete is relatively stable when the concrete age is 90 days, the resistivity of the solid fraction changes with time and can be expressed based on the electrical resistivity of concrete at 90 days:
  5 $${\rho }_{s}={\rho }_{90}\cdot \left(\left({x}_{1}{T}^{3}+{x}_{2}{T}^{2}+{x}_{3}T+{x}_{4}\right){\text{ln}}\left(t\right)+{x}_{5}{T}^{3}+{x}_{6}{T}^{2}+{x}_{7}T+{x}_{8}\right),$$
  where $t$ is the relative time based on the 90-day age (that is, when the curing time is 90 days, t = 1) and ${x}_{1}$, ${x}_{2}$, ${x}_{3},$ ${x}_{4},$ ${x}_{4},$ ${x}_{4},$ ${x}_{4},$ are the parameters to be solved.
3. The parameter $\eta$ is dimensionless and used to measure the proportion of series part and parallel part of solid phase and water–gas mixture phase in the concrete. Considering that the basic variables in the model are temperature and curing age, the parameter $\eta$ can be expressed as a function of temperature *T* and time $t$. Because the relationship of $\eta$ and two basic variables cannot be known advance, the commonly used exponential form is used for fitting. Thus:
  6 $$\eta ={y}_{1}\cdot {e}^{{y}_{2}T+{y}_{3}t},$$
  where $T$ is the temperature in Celsius and ${y}_{1}$, ${y}_{2}$ and ${y}_{3}$ are the parameters to be solved.
4. The parameter $\alpha$ is used to measure the proportion of series part and parallel part of water and gas in the pores. Similar to parameter $\eta$, $\alpha$ can be expressed as a function of time t and temperature as follows:
  7 $$\alpha ={\text{z}}_{1}{e}^{{\text{z}}_{2}T+{z}_{3}t},$$
  where ${z}_{1}$, ${z}_{2}$ and ${z}_{3}$ are the parameters to be evaluated. By using the test data in Fig. 6 and the least squares method to define the parameters to be determined in the concrete resistivity formula, the parameters can be obtained, as shown in Table 2.

**Table 2:** Parameters of the concrete resistivity model.

Parameter	${x}_{1}$	${x}_{2}$	${x}_{3}$	${x}_{4}$	${x}_{5}$	${x}_{6}$	${x}_{7}$	${x}_{8}$	${y}_{1}$	${y}_{2}$	${y}_{3}$	${z}_{1}$	${z}_{2}$	${z}_{3}$
Value	2.31	− 10.24	13.0	3.0	− 0.058	1.23	0.53	42.0	1.0	0.0	0.0	0.0	1.0	0.1

**Figure 7:**
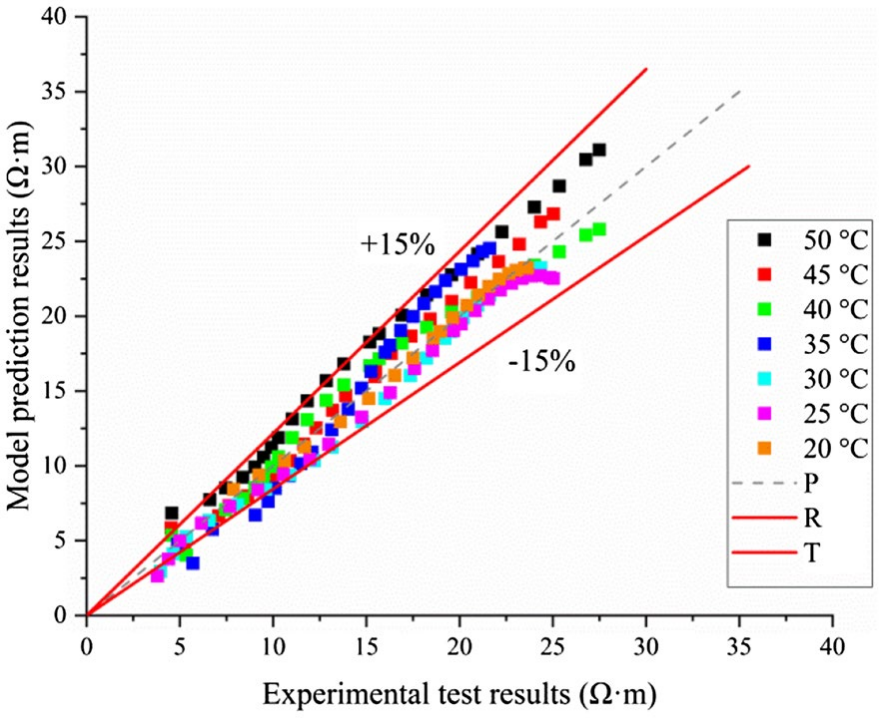
Comparison of the predicted and measured values of the proposed resistivity model.

To verify the prediction capability of the proposed resistivity model for concrete at different temperatures and curing times, the calculated results of the proposed resistivity model are compared with the measured results. The comparison is shown in Fig. 7.

As shown in Fig. 7, the deviation between the predicted and actual test values of the proposed resistivity model is within 15%, which verifies the accuracy of the proposed model and its prediction capability. This resistivity model can be used to determine the concrete electrical resistivity at any curing time and under different temperature conditions. Therefore, the equivalent age can be obtained by setting the development process of concrete resistivity under standard curing conditions as the reference and interpolating the concrete resistivity under nonstandard curing conditions onto the standard curing curve. For example, the electrical resistivity of concrete curing under standard conditions is ${\rho }_{1}$, and the equivalent age of concrete equals the age of concrete ${t}_{1}$. Similarly, when the electrical resistivity of concrete curing at 30 °C reaches ${\rho }_{1}$, the age of concrete is ${t}_{2}$. Because the electrical resistivity of concrete equals each other for the concrete curing under standard conditions and nonstandard conditions, we can consider the concrete to have reached the same state; that is, the equivalent age of the concrete curing in different conditions is the same.

For concrete with the same age under different curing conditions, the equivalent age of the concrete can be obtained by the equivalent coefficient. The equivalent coefficient can be calculated by the concrete electrical resistivity model. For example, given that the curing temperatures of the two samples are 30 °C and 20 °C and the age of concrete is 5 days, the corresponding resistivity is ${\rho }_{30}$ and ${\rho }_{20}$ respectively. The equivalent coefficient for the equivalent age is as follows:

$$\upzeta =\frac{{\rho }_{30}}{{\rho }_{20}}=\frac{22.32}{20.33}=1.10.$$

For the 5-day concrete at 30 °C, the modified electrical resistivity of the concrete is $22.32\times 1.10=24.55$ Ω m. Then, the equivalent age is obtained by interpolating the equivalent resistivity on the standard curing curve (Fig. 4). Therefore, the equivalent age of the concrete curing in 30 °C is 8.4 days.

### Comparison with other equivalent age methods

The environmental temperature may be constant or variable in the process of increasing concrete strength. The reference temperature in the equivalent age model is the standard curing temperature of 20 °C. Age equivalent results are compared with those of the accepted equivalent method in the specifications for hydraulic concrete construction (DL/T5144-2015), which was developed based on the Nurse-Saul equation^55^. The national standard provides the following formula for calculating the equivalent concrete age during quality inspection:

8 $$t_{T} = \sum\limits_{i = 1}^{n} {\Delta t_{i} } \alpha_{i}$$

**Figure 8:**
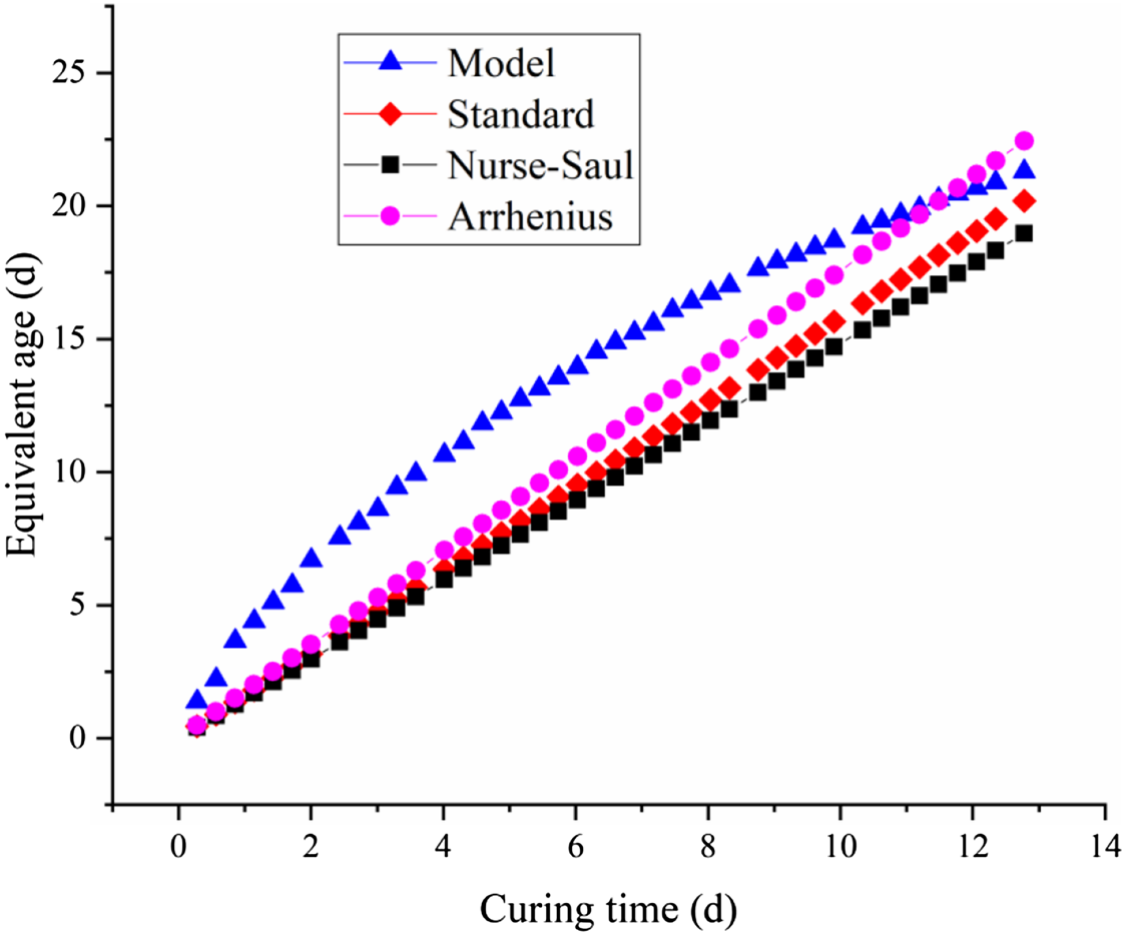
Comparison of equivalent age by different methods under constant-temperature curing conditions.

To obtain the equivalent age, the temperature under the constant-temperature curing conditions is set to 30 °C, and the curing time is set to 12 days. The results of equivalent age obtained from the national standard and those in accordance with the equivalent age calculated from the resistivity model are drawn on the same coordinate plane, and the results are shown in Fig. 8.

As shown in Fig. 8, the equivalent age of concrete specified in the national regulation and Nurse-Saul method show a linear increase, whereas the equivalent age calculated by the proposed resistivity model and Arrhenius equation show a certain nonlinear change. The equivalent coefficient of national regulation and Nurse-Saul is a constant at fixed temperature, so the equivalent age of concrete obtained by these two methods increases linearly. Arrhenius equation includes the parameter of concrete apparent activation energy, which is usually affected by temperature, so the equivalent age obtained by Arrhenius equation is a nonlinear change process in theory. However, it is often difficult to obtain the variation law of concrete apparent activation energy with temperature, which varies with type of concrete. In this study, the value of apparent activation energy commonly used for ordinary concrete is taken as 41,752 J/mol.

The concrete equivalent age calculated by the proposed method increases rapidly in the early stage of hydration. Then, the growth rate becomes progressively slower, but the overall trend is still a gradual increase. This phenomenon can be explained by the rapid reaction rate of concrete in the initial stage of mixing, and the physical properties of various aspects, such as the strength, also increase rapidly. Therefore, the growth rate of the equivalent age is high in the early stage. As the cement solidifies and hardens, the amount of cement clinker involved in the reaction decreases. In addition, many cement hydration products are generated, resulting in some cement clinker being “isolated”. Therefore, the overall reaction rate shows a gradual trend; at this time, the growth rate of concrete age is slow. Analysis of the reaction rate of the concrete shows that the phase growth process and the increasing electrical resistivity process are consistent with the hydration reaction rate of coagulation. The physical properties of concrete are closely related to the degree of hydration of the reactants. Therefore, the equivalent age of concrete based on the proposed resistivity model is reasonable, as this model can consider the effects of the reaction rate. The effective curing time of concrete in this study is 0–12 days due to limitations in testing time and conditions. During this period, the growth rate of the concrete age initially increases and then decreases.

Under variable-temperature curing conditions, the temperature changes continuously. When obtaining the equivalent concrete age, the midpoint of each temperature curve section is taken as the curing temperature, and the curing temperature is considered to be a constant value. For spacing, temperature projection increases gradually by 1 °C. The temperature history during curing is shown in Table 3.

**Table 3:** Evaluation of temperature under the variable-temperature curing conditions.

Temperature (°C)	22	24	22	20	21	22	23	24	26	27	28	29	30	31	32
Duration (h)	10	15	5	26	18	12	23	26	13	10	28	13	18	16	20
Cumulative time (days)	0.4	1	1.2	2.3	3	3.6	4.5	5.6	6.2	6.6	7.7	8.3	9	9.7	10.5

**Figure 9:**
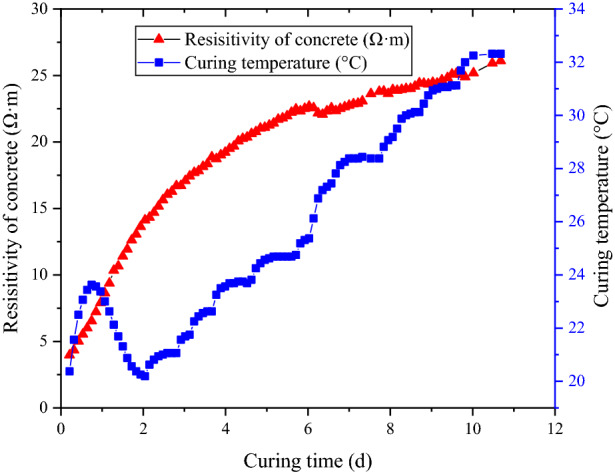
Curing temperature and concrete resistivity variation process under the variable-temperature curing conditions.

The concrete resistivity growth curve under the variable-temperature curing conditions is shown in Fig. 9.

**Figure 10:**
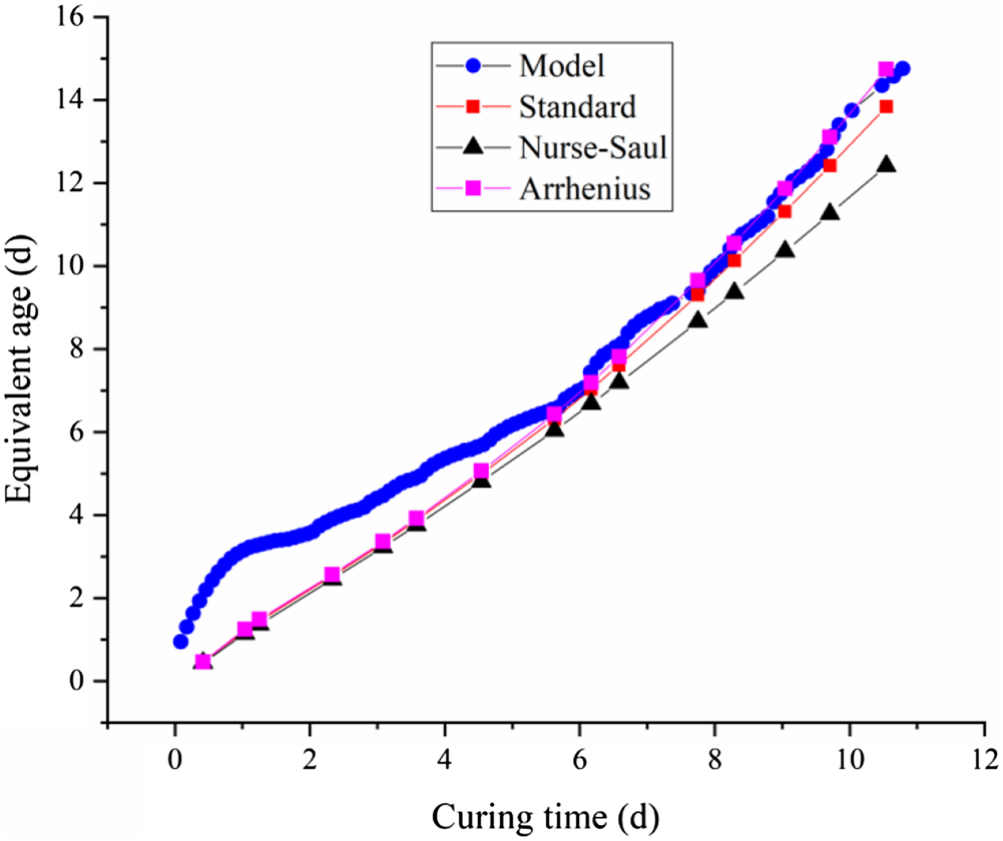
Comparison of the equivalent age obtained by different methods under the variable-temperature curing conditions.

A comparison of the equivalent age results calculated by the electrical resistivity model and the equivalent results obtained by the standard method, Nurse-Saul and Arrhenius equation is shown in Fig. 10.

As shown in Fig. 9, because the curing temperature changes continuously, a period of time near a relatively stable temperature can be regarded as the stable period of the temperature. According to the equivalent result (Fig. 10), under the variable-temperature curing conditions, the results of the proposed resistivity model, standard method and Arrhenius equation increase nonlinearly in general. When the curing time less than 6 days, the equivalent age obtained by the resistivity model is larger than that obtained by the other three methods. While, when the curing time more than 6 days, the equivalent age obtained by the proposed model is close to that obtained by standard method and Arrhenius equation. In reality, the age growth process of concrete is a nonlinear process that is influenced by the environmental temperature. The equivalent results from the proposed resistivity model are affected by the temperature change. When the temperature increases, the growth rate of the concrete equivalent age accelerates; when the temperature decreases, the growth rate of the concrete equivalent age slows. The results of the proposed method are consistent with those of the standard method and Arrhenius equation to some degree, the difference mainly exists in the early age stage. One of the possible reasons for this phenomenon is that the resistivity of concrete ican reflect the reaction rate of concrete to a certain extent, which avoids the difficulty of solving the apparent activation energy in Arrhenius equation. Therefore, the proposed method considers the influence of temperature more flexibly. The influence of temperature on the development of the equivalent age in different stages is also considered. For example, the increase rate of the equivalent concrete age at 30 °C in the early stage is different from that in the late stage. The influence of the temperature history on the equivalent age of concrete is recorded in the changes in resistivity.

## Discussion and conclusion

A concrete equivalent age method based on concrete electrical resistivity is proposed. The method is implemented by measuring concrete resistivity and modifying the effect of temperature using the resistivity development curve under standard curing conditions. To achieve the equivalent concrete age, this study has designed an experimental test system that automatically measures concrete resistivity, establishes a resistivity model based on Winner bounds, solves the parameters in the proposed resistivity model, and applies the model to concrete age equivalence. The experimental test system is developed based on the two-electrode method, and the two directions of the concrete electrical resistivity are measured to calculate the average value of the concrete to eliminate the effects of the polarization phenomenon as much as possible.

For the equivalence of the concrete age under different temperatures, the equivalent coefficient can be obtained by the electrical resistivity model. It should be noted that the parameters of the electrical resistivity model are only suitable for the concrete used in this paper. The parameters should be recalculated for concretes with different compositions. Using the proposed resistivity model, the equivalent age of concrete is achieved under the variable-temperature curing conditions. By solving the parameters in the proposed resistivity model, the functional relationship between resistivity and curing time and temperature is established. The temperature of concrete resistivity is modified, and the equivalent age is obtained in accordance with resistivity data under standard curing conditions. Compared with standard method, Nurse-Saul method and Arrhenius equation, it is found that the proposed model has strong applicability to constant temperature and variable temperature curing conditions, and can fully consider the nonlinearity of concrete age growth. Concrete resistivity itself can reflect the change of concrete activation energy to a certain extent. Therefore, the equivalent age obtained by resistivity equivalence is relatively closer to the actual situation and more flexible, especially for early age concrete.

The proposed method has good feasibility and can be used to monitor the development of concrete age quickly and in real time, and because of the relationship between temperature and concrete resistivity, the development process of equivalent concrete age under complex temperature changes can be considered. The proposed method has better adaptability and higher accuracy in determining the equivalent concrete age than the standard method under variable-temperature curing conditions. In addition, the proposed method is nondestructive and can be repeated many times on one sample, especially under complex temperature change conditions. However, it should be noted that the concrete used in this paper is actually cement mortar without coarse aggregate, because the concrete with coarse aggregate has poor electrode contact and non-uniformity, which cannot solved present. In the future, we will conduct further research on concrete samples.
